# Sustaining village malaria worker programmes with expanded roles: Perspectives of communities, healthcare workers, policymakers, and implementers in Vietnam

**DOI:** 10.1371/journal.pgph.0003443

**Published:** 2024-08-06

**Authors:** Hue Nguyen, Monnaphat Jongdeepaisal, Duong Anh Tuan, Panarasri Khonputsa, Thang Ngo, Christopher Pell, Marco Liverani, Richard J. Maude

**Affiliations:** 1 Faculty of Tropical Medicine, Mahidol-Oxford Tropical Medicine Research Unit, Mahidol University, Bangkok, Thailand; 2 Nuffield Department of Medicine, Centre for Tropical Medicine and Global Health, University of Oxford, Oxford, United Kingdom; 3 National Institute of Malariology, Parasitology and Entomology, Ministry of Health, Ha Noi, Viet Nam; 4 Amsterdam Institute for Global Health and Development (AIGHD), Amsterdam, The Netherlands; 5 Department of Global Health, Amsterdam University Medical Centers, Academic Medical Center, Amsterdam, The Netherlands; 6 Centre for Social Science and Global Health, University of Amsterdam, Amsterdam, The Netherlands; 7 Department of Global Health and Development, London School of Hygiene and Tropical Medicine, London, United Kingdom; 8 School of Tropical Medicine and Global Health, Nagasaki University, Nagasaki, Japan; 9 Faculty of Public Health, Mahidol University, Bangkok, Thailand; 10 The Open University, Milton Keynes, United Kingdom; Tulane University School of Public Health and Tropical Medicine, UNITED STATES OF AMERICA

## Abstract

**Background:**

In Vietnam, multiple types of community-based malaria workers are recruited to promote access to malaria testing and treatment for at-risk mobile and migrant populations. However, as the country approaches elimination, these roles are at risk from declining investment. This article characterises the different types of workers and relevant health policy in Vietnam, and explores stakeholder perspectives on sustaining and expanding the roles of these workers in the malaria elimination context.

**Methods:**

We conducted a rapid policy document review to describe the policy background of community-based health care in Vietnam and identify key informants. In-depth interviews were conducted with policymakers and implementers (n = 11) in different government sectors, international, and civil society organizations. We also conducted interviews (n = 8) and two focus group discussions with community members (n = 12), and interviews with health workers (n = 9) in 18 communities in Phu Yen and Binh Thuan provinces in the central region.

**Results:**

Our study identified three main types of malaria workers: village health workers (VHWs), malaria post workers (MPWs) and other groups of workers supported by civil society organisations (CSO). Workers reported being willing to maintain their current roles and potentially provide additional services that respond to community needs, although they were concerned about excess workload and inadequate income. Besides working in a variety of jobs to secure their incomes, mainly in agriculture, VHWs in particular were primarily tasked with supporting the delivery of a wide range of health services from commune health stations. Faced with reduced patients, MPWs and CSO-supported workers could be tasked with the following potential roles: case notification for infectious diseases, real-time data collection and reporting, and screening for non-malaria illnesses using other rapid test kits. There was agreement that the community-based health network is crucial to health care delivery in remote communities and to ensure comprehensive access to care among vulnerable populations. However, policymakers and implementers stressed concerns about their limited skillsets, the inadequate budget to support these workers, and the regulation constraining them from performing diagnosis and treatment activities, highlighting the priority to maintain the capacity of workers and funding allocation through provincial advocacy and multi-programme collaboration.

**Conclusions:**

This study highlights the crucial role of community-based health workers in eliminating malaria in Vietnam. Sustained efforts are needed to maintain accessible case detection and treatment while addressing local health concerns beyond malaria. Implementing these strategies requires prioritizing the mapping of groups most in need and developing enablers to raise community awareness and maintain the capacity of these workers. Ensuring political advocacy, financial resources, and coordination between multiple groups are key to maximizing impact and integrating malaria activities into the broader health system.

## Introduction

Although progress towards malaria elimination has been made in the Greater Mekong Subregion (GMS), the disease still poses a significant risk in some areas [1]. In remote rural settings, where healthcare access is limited, the impact of the disease is especially severe, with the heaviest health and economic burden on affected communities [2]. A significant number of these communities face shortages of healthcare professionals and inadequate infrastructure [3]. To effectively target populations at risk, it is crucial to establish accessible malaria services in communities. The introduction of rapid diagnostic tests (RDTs) for malaria in the early 2000s played a significant role in expanding the responsibilities of village malaria workers (VMWs) to ensure timely diagnosis and treatment in the community [4,5].

The primary strategy for eliminating malaria is the early detection and treatment with effective drugs. In Southeast Asia, this relies heavily on an active VMW programme. However, as malaria cases decrease, there will be a relative increase in non-malarial febrile illnesses. Because VMWs are currently equipped only to test for and treat malaria, their inability to address other causes of fever could jeopardize the VMW network’s sustainability. Weakening VMW networks poses a significant threat to the rapid elimination of malaria. Additionally, with tremendous progress towards malaria elimination over decades in this subregion, it’s crucial to ensure long-term effectiveness of malaria responses, especially given the reduced funding from external donors. Countries have thus explored integration of malaria programmes into robust national health systems [6].

In Vietnam, there has been a large reduction in cases from 2000 onwards [7]. The remaining transmission areas are primarily located in hilly, forested areas with the most at-risk groups including community members engaged in forest farming, ethnic groups in forest settlements, forest workers and seasonal mobile workers in agriculture and construction, and patrolling officials such as military, border police, and wildlife protection units [8]. These varied groups of at-risk populations require tailored malaria control strategies, especially among those visiting forested areas overnight with limited use of personal protections and marginalized groups with limited access to local infrastructure and health service coverage.

In malaria-endemic provinces, there are specialized staff serving as focal points at provincial, district and commune levels [8,9]. At the community level, the health response relies heavily on commune health staff and volunteer village health workers (VHWs), who support malaria-related activities alongside midwifery and public health programmes, such as methadone therapy, HIV prevention, and reproductive health [10]. Their function and duties have been officially regulated since 1999 [11]. Furthermore, depending on the stage of malaria control and elimination, additional community-based malaria cadres, referred to as malaria post workers (MPWs), have been established to increase the accessibility of malaria services for hard-to-reach populations in high transmission areas [12]. To address the needs of these population groups, tailored interventions were designed to strengthen malaria case management services, including provision and supervision of diagnosis and treatment, by commune health staff, MPWs, and in some areas, army health posts [13]. Nationally, there were approximately 9,000 community-based health workers providing malaria-related activities including VHWs and MPWs in 2022.

With the country approaching elimination, the national malaria programme emphasized the essential role of the grassroots level in the detection and treatment of malaria cases [13,14]. There is a risk of reduced funding leading to a scaling back of services, which will hamper elimination efforts. To maintain community-based malaria services, one strategy could be to broaden the responsibilities of MPWs and other malaria workers within the civil society organization (CSO) network and programmes. Identifying new roles and activities will keep them relevant and attract more people with fever to seek help from the workers. In low transmission areas, a larger group of VHWs could be leveraged to deliver additional malaria services, ensuring the continued early diagnosis and treatment of malaria; the strategy was also proposed in the 2021–2025 National Strategic Plan [8].

However, to successfully implement this approach, it is crucial to elucidate the policy challenges and opportunities at various levels of the healthcare system. Currently, there is limited understanding of how expanding the roles of these workers aligns with Vietnam’s elimination priorities, the existing public health requirements in malaria-endemic communities, and how these workers can be effectively utilized to achieve these objectives.

This article investigates the potential for, and obstacles associated with, sustaining and expanding the roles of these malaria workers by drawing insights from stakeholder analysis, in-depth interviews (IDIs) and focus group discussions (FGDs) with stakeholders, including policymakers, implementers, healthcare personnel, and members of malaria-endemic communities in Vietnam.

## Methods

### Research project and study design

This study was part of a wider multidisciplinary multi-country research project supported by the Global Fund to Fight AIDS, Tuberculosis and Malaria entitled *Sustaining village health worker programmes with expanded roles in the Greater Mekong Subregion* (2021–2023). The project aimed to inform ongoing efforts across the GMS to sustain malaria workers and accelerate the elimination of *Plasmodium falciparum* malaria in the subregion [15]. It comprised an Asia-Pacific wide systematic review, scoping survey, and interviews of National Malaria Control Programmes (NMCPs) and implementing organisations [16]. For the GMS countries, qualitative studies comprising in-depth interviews and focus group discussions were conducted in Cambodia, Thailand, and Vietnam; the results from Vietnam are reported here and published separately for the other two countries.

This study is a prospective policy analysis that seeks to understand the contextual opportunities and challenges of policy regarding malaria workers within a health system [17]. Policy document review and stakeholder analysis were performed to identify key informants and policy-related issues [18]. We used a qualitative descriptive design to explore inductively the prospects for role expansion among village malaria workers. Qualitative research methods, including interviews and focus groups, were used to gain an in-depth understanding of the topic in individual and group settings. In line with this approach, we aimed to uncover themes from the qualitative data, using a codebook that was developed as they emerged.

### Vietnam’s national malaria control programme

The national malaria control programme in Vietnam, the National Institute of Malariology Parasitology and Entomology (NIMPE), and two regional Institutes of Malariology Parasitology and Entomology (IMPE), are responsible for nationwide technical direction of malaria control activities [19]. At the local level, Provincial Centers for Disease Control (PCDC) under supervision of the Department of Health are responsible for all malaria activities [11]. This has resulted from a significant decentralization of Vietnam’s healthcare system in recent years with the implementation of the Law on Health Insurance (2014) and the Law on Medical Examination and Treatment (2009) [20,21]. Since 2008, the National Assembly has mandated that provinces allocate at least 30% of their budgets to preventive health measures, which includes funding for malaria-related activities [22]. In 2017, the Prime Minister’s Decision 1125/QD-TTg led to the termination of national funding for malaria programmes at the provincial level, and provinces securing their own budgets with support from the Global Fund and other donors to implement the programmes from 2021 onwards [23]. Under the new system, local departments are given more authority to manage the healthcare systems at provincial, district, and commune levels and implement their own healthcare policies and strategies with more control over healthcare financing and management.

In addition, under Vietnam’s Social Health Insurance Scheme, 95% of healthcare expenses related to malaria examination, diagnosis, and treatment are covered for patients with a Health Insurance Card, leaving the patients to co-pay 5%. Such diagnosis and treatment are provided free-of-charge if the patients are identified as eligible based on their socio-economic status. Beyond this scheme, health facilities that are supplied with microscopes, commodities, rapid diagnostic tests, and medicine from external funds are able provide free treatment to their patients [24].

### Settings

**Fig 1:**
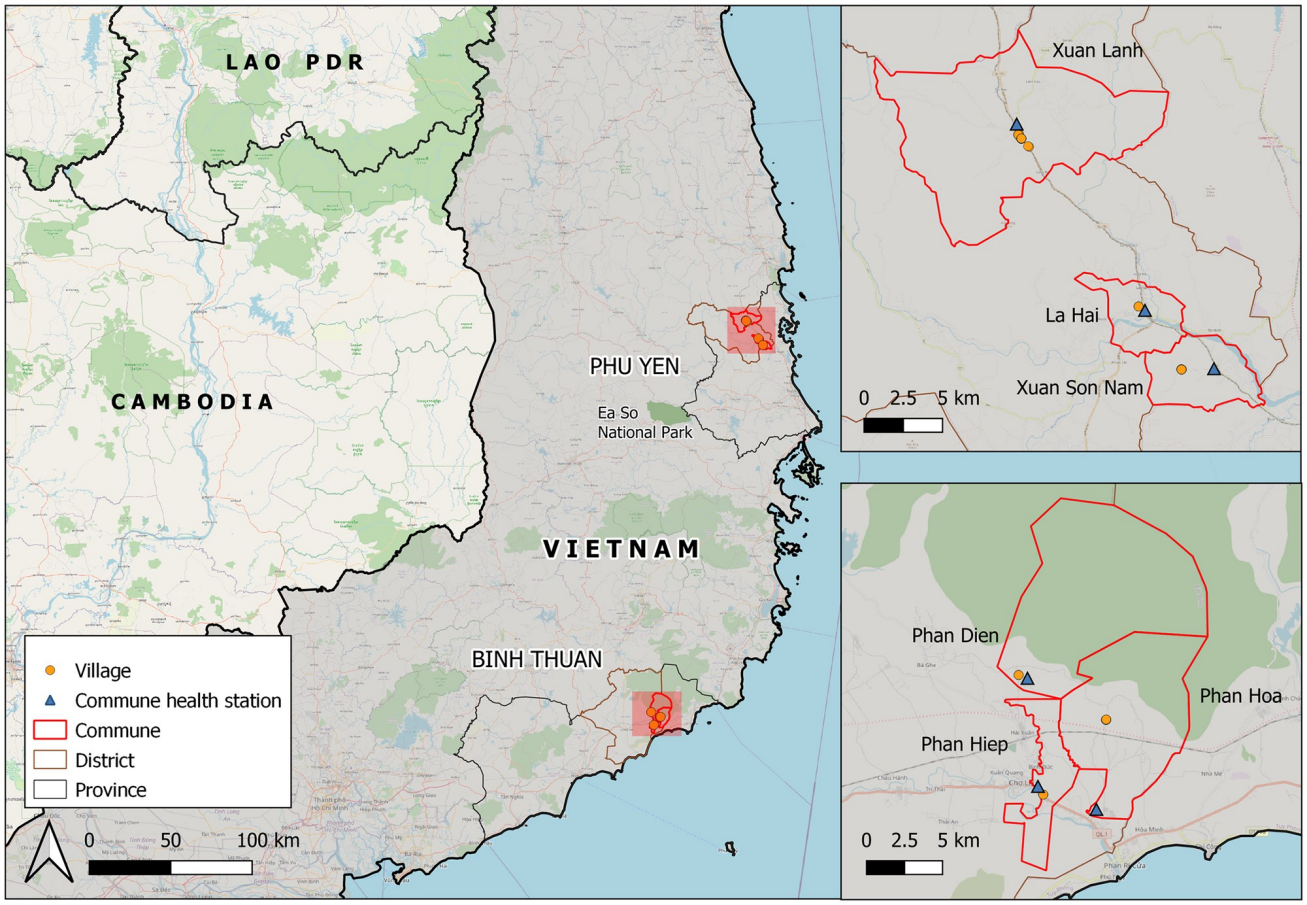
Study sites and locations of study villages in southeast Vietnam. The map was created using QGIS software version 3.26 (Buenos Aires) and contains information from OpenStreetMap and OpenStreetMap Foundation, which is made available under the Open Database License. National and provincial administrative boundaries from Global Administrative Areas version 3.6 (https://gadm.org/download_country_v3.html). Ea So Park boundary from Open Development Mekong (https://data.opendevelopmentmekong.net/dataset/national-protected-areas-in-vietnam).

Locations for community interviews and focus group discussions were selected in consultation with NIMPE. Binh Thuan and Phu Yen provinces in the central-southern region were selected as low and higher transmission settings and because of feasibility of data collection (see Fig 1). These included three communes (Phan Hiep, Phan Hoa, and Phan Dien) in Bac Binh district, Binh Thuan; and 3 (La Hai, Xuan Lanh, and Xuan Son Nam) in Dong Xuan district, Phu Yen. According to the 2021 census, Binh Thuan had a population of 1,246,310 and Phu Yen of 875,540 [25]. These were two of the ten highest malaria endemic provinces from 2016 to 2020 [19], with average annual numbers of 263 and 139 cases in Phu Yen and Binh Thuan respectively [26]. The national malaria incidence rates per 1000 population were 0.11, 0.09, 0.07, 0.06, and 0.02 during 2016–2020.

According to the National Strategic Plan for Malaria Control and Elimination 2021–2025, Binh Thuan was set to achieve elimination early in 2018 and Phu Yen in 2024 [8]. When planning this study in 2021, Binh Thuan was close to malaria elimination and moving towards the prevention of re-establishment phase. Some mountainous forested areas in Phu Yen remained endemic but forest activities in the province have been minimal recently due to stricter enforcement restricting visits to Ea So Nature Reserve Forest, which borders the other two endemic districts in Dak Lak and Gia Lai provinces in the west, raising the concern of importation to Phu Yen. Additionally, COVID-19 travel restrictions had reduced local movement and visits from other provinces.

In the study communes, the majority of the residents spoke Vietnamese, and some Cham, the language spoken by the local Champa population in the central region. The main livelihood activity is farming fruits and rice, and paper trees. The majority of the farmers were hired locally by landowners to work in rice fields and plantations; some could also be temporarily hired to collect wild products in the forest.

### Respondents and recruitment

In the initial phase, the research team consulted with PCDC and respective CHS to identify high risk communes in the provinces and community members’ access to the health stations. FGDs were conducted with community members and IDIs with community members and health workers. Recruitment for IDIs and FGDs at the study sites was planned and coordinated with leaders of the CHS who informed potential respondents about the research topic and the purposes of the interviews and focus groups. They were contacted either in-person or by telephone call.

Adult community members (>18 years old) were invited to either join the one-on-one interviews or the focus group discussions. They were purposively identified through CHS in target villages and endemic communes. Diversity of ages and gender were the main selection consideration for community members. The interviews and focus groups intended to elicit the experience and perceptions of the communities regarding common illnesses and access to care for malaria and from VHWs and/or MPWs in their communities. The one-on-one interviews were designed to draw out in-depth information from the respondents, while the focus groups explored further their expectations of future malaria care in their communities in a dynamic and open setting.

Purposive sampling was used to recruit potential VHWs and MPWs for in-depth interviews. They were selected based on their role in delivering malaria-related services in target communities: MPWs were tasked to perform malaria testing, treatment, control, and/or prevention activities in endemic areas; VHWs were trained and tasked to assist CHS in providing primary health services, and in endemic areas also assist in malaria-related activities. According to the health statistics yearbook in 2018, 96.9% of villages had active VHWs in Binh Thuan and 100% in Phu Yen [27].

For policymaker and implementers of malaria programmes, we conducted a desk review of the current public health system in Vietnam to map and identify policymakers and other relevant informants to engage in in-depth interviews. The Ministry of Health, international organizations (IOs) and civil society organizations (CSOs) were all actively contributing to the malaria elimination goal. In consultation with NIMPE, we purposively selected representatives from different government and non-government organisations from central and local levels. In addition, at the end of each interview, a snowball sampling method was used by asking respondents to suggest other policymakers and implementers to be involved in this study. They were invited to join the interviews by professional email contact or formal invitation letter as representatives of their organisations.

### Data collection tools

Due to the exploratory nature of this study, IDI guides were designed to be loosely structured and tailored to the specific role and expertise of each informant. The interviews aimed to gather perspectives and expectations regarding prospective community-based health services and the necessary resources for effectively implementing expanded services. The FGDs provided a group setting to explore local health concerns and how expanded roles of malaria workers could be implemented and developed to better serve specific population groups within the communities. This included topics such as patient referral and transportation, as well as the work conditions for the workers. The development of the IDI and FGD guides was based on the results of a rapid review and a previous study on forest malaria and at-risk groups in Thailand [28]. Initially drafted in English, the guides were translated into Vietnamese and reviewed for clarity and accuracy (see S1 Appendix for IDI and FGD guides). These guides were refined as new findings emerged during data collection, for example, the guides were used flexibly to guide conversations and were updated based on insights gained from the interview responses, including resource management for different types of workers, capacity building and development for workers, and COVID-19 outbreak related challenges and lessons learnt.

### Data collection

Qualitative data collection activities were conducted during March and July 2022 by a female Vietnamese researcher (HN), and male NIMPE epidemiologist (DAT), with the support of in-person and online interviews by two researchers (ML and MJ). At the study sites, interviews and focus group discussions were conducted at nearby CHS, villages houses, or malaria posts in the communes. Interviews with policymakers and implementers were conducted at their places of work, in-person or online. Field researchers (HN and DAT) were trained in field data collection regarding good clinical practices and public health issues and sought their informed consent prior to the interviews or focus group discussions. The interviews were conducted mostly one-on-one by the main interviewer (HN) who introduced herself and the reasons for interests in the topic to the respondents. Respondents were also provided with a participant information sheet that informed them of the research objectives and data confidentiality issues. All focus group discussions and interviews were conducted in Vietnamese and/or English and audio recorded, with an average of 45 minutes duration. Observations, debrief sessions, and consultation meetings with communities and local stakeholders over a three-week data collection period at the site were recorded in detail with fieldnotes in Vietnamese and English by the study team. At the end of the interviews and the group discussions, respondents were asked if they would like to correct any prior responses or further comment on any particular issues.

Clearance was sought and received from respective PCDCs and district health centres (Bac Binh in Binh Thuan and Dong Xuan in Phu Yen), and 6 commune health stations (3 in each province) prior to data collection. The study team was accompanied by PCDC staff during data collection in the communes, and met with the directors of the district health centres on the first and last days of data collection to provide debriefs on the data collection process and receive feedback on the findings.

### Data analysis

Audio recordings were transcribed verbatim in Vietnamese and/or English depending on the language used during the interviews (HN and/or ML). The transcripts were imported into NVivo version 13 (QSR International) for qualitative thematic analysis [29]. Every transcript was deductively coded by the main Vietnamese researcher (HN) using the codebook initially developed for the related study in Thailand by the Thai researcher (MJ), depicting the following key themes: livelihoods, health concerns, access to care, work experience, opportunities, challenges, and recommendations. As the coding process progressed, sub-themes that surfaced from the data were inductively integrated into the codebook. The study team consulted on data saturation after the first 25 interviews were conducted. In order to gain a deeper understanding of the various stakeholders, their resources, and their perspectives on the expansion of roles, initial outputs such as aa stakeholder matrix table and implementer network map were created [18,30] to visualize the roles played by different stakeholders and facilitate a comprehensive analysis. During the analysis and reporting period, the preliminary and final results were presented to NIMPE for validation and feedback.

### Ethics statement

Ethical approval was obtained from the Oxford Tropical Research Ethics Committee (OxTREC Reference: 535–21 dated 24 August 2021 and 552–21 dated 22 September 2021) and Institutional Review Board (IRB) at NIMPE (approval number 35-2021/HDDD, 22 December 2021). All respondents provided written informed consent to join the study. Those who agreed to join the interviews and/or the group discussions were asked for their consent to be audio-recorded. All the respondents agreed to join the study and were informed that they can withdraw from the study without penalty at any time. Additional information regarding the ethical, cultural, and scientific considerations specific to inclusivity in global research is included in the (S1 Checklist).

## Results

Based on desk reviews of literature and policy documents [8,19,26,31,32], the results of stakeholder identification and network mapping activities are shown in Table 1, reflecting the current structure of malaria programme implementation and stakeholders. Policymakers and implementers of malaria-related activities function at multiple levels. Alongside the national malaria programme, two departments under the Ministry of Health oversee regulations regarding medical services for various health programmes in the country. Government units performing functions of implementing malaria activities exist at provincial, district, and commune levels. Various international organisations (IOs) provide technical and financial assistance to the national programme and the implementing civil society organisations (CSOs) in the country. We were able to identify five cadres of community-based workers that perform malaria-related activities in Vietnam. Alongside government-managed VHWs and MPWs, CSOs recruited and supervised other types of workers, such as malaria elimination volunteers (MEVs), community malaria action teams (CMATs), and mobile outreach teams (MOTs) to perform active malaria tasks targeting mobile and migrant populations. This complex network of the malaria programme implementers and cadres of workers is further elaborated in S2 Appendix.

**Table 1:** Stakeholder identification and mapping; * identifies IDI respondents.

Government within the Ministry of Health		International organisations
General health system	Vertical programme	
**Central level** General Department of Preventive Medicine (GDPM)* Department of Medical Service Administration (DMSA)	National Institute of Malariology Parasitology and Entomology (NIMPE)* Institute of Malariology Parasitology and Entomology (IMPE) Quy Nhon and Ho Chi Minh City	Bill and Melinda Gates Foundation (BMGF) Clinton Health Access Initiative (CHAI)* PATH* Population Service International (PSI) President’s Malaria Initiative (PMI)-USAID World Health Organization (WHO)
**Sub-national level** within the Department of Health (DoH) Provincial Center for Disease Control (PCDC)* District Health Center (DHC) Commune Health Station (CHS)		**Civil society organisations** Center for Supporting Community Development Initiatives (SCDI)* Health Poverty Action (HPA)* Vietnam Public Health Association (VPHA)
Village health worker (VHW)*	Malaria post worker (MPW)*	CSO-supported workers

At-risk population and community in malaria endemic areas*.

### Demographics of respondents

We interviewed 28 respondents in total: policymakers and implementers (n = 11) at national and sub-national levels, VHWs and MPWs (n = 9), and community members (n = 8) in Binh Thuan and Phu Yen provinces. Two FGDs were conducted with community members in each province with a total of 12 participants. Table 2 shows the general characteristics of participants in this study. Of policymakers and implementers involved in the study, most (8/11) were male. Of community members and health workers, two thirds (20/29) were female. The average duration of work experience among stakeholders was 14.7 ± 8.7 years and for the health workers 9.1 ± 8.5 years. Six out of nine VHWs and MPWs achieved the highest level of education at a vocational school. The numbers of households the health workers oversaw varied from 356 to 1,039 depending on the population size in their villages.

**Table 2:** Demographic characteristics of IDI and FGD respondents; with percentages of total respondents (n = 40) reported in brackets.

Types of respondents	Community members (n = 20)	VHWs and MPWs (n = 9)	Policymakers and implementers (n = 11)
Data collection method			
IDI	8 (20)	9 (23)	11 (28)
FGD	12 (30)	-	-
Gender			
Male	7 (18)	2 (5)	8 (20)
Female	13 (33)	7 (18)	3 (8)
Age (years)			
20–29	1 (3)	1 (3)	0
30–39	4 (10)	4 (10)	4 (10)
40–49	8 (20)	0	2 (5)
50–59	5 (13)	3 (8)	5 (13)
60 and over	2 (5)	1 (3)	0
Experience (years)			
Mean	-	9.1	14.7
SD	-	8.5	8.7
Min	-	<1	3
Max	-	22	28

Out of 11 representatives of government and non-governmental organisations, seven were key implementers in malaria elimination programmes and policy makers for grassroots health development strategies at central (n = 5) or local level (n = 2). Four respondents were representatives of CSOs and international organizations (IOs) directly implementing malaria-related activities at the community level. Eight were in management positions or boards of directors, whereas three were technical advisors or specialists in the programmes.

The emerging themes were identified from investigation and organized according to the three following domains, with illustrative quotations from the interviews and focus groups:

1. Community-based health workers in delivering malaria services: roles, motivations, and performance;
2. The prospects of sustaining their roles and community-based malaria activities;
3. Potential barriers and enabling factors of role expansion.

### Community-based health workers in delivering malaria services: Roles, motivations, and performance

#### Roles and services

The community-based malaria workers, whose locations are determined by local malaria epidemiology, were identified as an extension arm of the public health system in connecting hard-to-reach populations with malaria services. Here, we present the findings related to their roles, motivations, and performance. The interviewed workers included eight VHWs and one MPW in the two provinces. The CSO-supported volunteers were not present at their districts; however the programme management and/or their supervisors were interviewed accordingly.

Malaria activities were performed by different groups of workers in the endemic areas, primarily depending on the transmission situation and at-risk population. In communities with low endemicity in Binh Thuan, all interviewed VHWs mentioned that they only provided malaria-related health promotion and referred suspected malaria cases to the CHS. Whereas in Phu Yen, VHWs and MPWs reported also being tasked with more complicated roles, such as collecting blood slides and/or conducting RDTs for the forest goers in their villages, in addition to providing information about malaria during their routine household visits.

*“When I started working as a village health worker*, *the CHS told me if I conducted a test*, *found a positive case of malaria and that person had symptoms of having the parasite*, *the first step was to notify the CHS*, *then the CHS would guide me in the next steps in detail to refer [the person] to the CHS*. *Or the CHS instructed the patient to get treatment there or referred them to the district hospital*.*”*

*(Female*, *Village health worker 5*, *Phu Yen)*

In high-transmission communes, additional workers were recruited and tasked with malaria-specific roles: MPWs and volunteers among the CSO-supported networks, including MOTs, MEVs, and CMATs. Recruitment criteria for these cadres were determined by the Global Fund-supported implementing organizations. The MPWs, MEVs and MOTs were required to have gained health-related qualifications and their roles included malaria case detection and notification; conducting RDTs, and collecting blood slides from forest goers. The CMATs were described as local influencers who motivate and help forest goers to access the nearest services. Within the endemic communities, which have both VHWs and community-based malaria cadres, the malaria-related tasks were usually performed by the latter groups. In Phu Yen, however, interviews with a MPW and a VHW in the same commune indicated that their malaria-related tasks overlapped, although they communicated to avoid duplication of activities with the same households and community members.

*“I don’t think we have any duplication in malaria-related activities*, *because he (VHW) is very busy*, *and he usually goes and conducts the tests when he gets some brief window of free time*. *I have more time in the day*. *Whenever I hear someone who recently travelled then slept in the forest with the high risk of getting malaria*, *I go there very soon to confirm the information with the neighbour then conduct the tests for suspected cases*. *I usually check with him (the VHW) before I go there [to the potential malaria positive case’s house]*.*”*

*(Male*, *Malaria post worker*, *In-depth interview*, *Phu Yen)*

At the sites, respondents indicated that VHWs and MPWs were unable to provide antimalarials; they were primarily tasked with facilitating case referral to health facilities. For MPWs, once a positive case was confirmed, the workers were additionally responsible for facilitating case investigation, case reporting, foci investigation and following up to monitor adherence to treatment and check for parasite clearance.

Except for VHWs, none of the other cadres were reported to perform roles beyond malaria-related activities. The VHWs, as a larger group of workers within the health system, performed roles that are related to general healthcare. Identified in the policy analysis, Circular 27/2023/TT-BYT [33] indicates that VHWs must complete a six-month training course and obtain a certificate to become qualified. At the sites, these included providing information on family planning, contraceptive choices, vaccinations, de-worming and vitamin A supplements for children. The focus group indicated how female community members were generally more aware of the services provided by VHWs compared to their male counterparts because they received information on vaccinations during their pregnancy from VHWs. Furthermore, VHWs were also likely to be females who were better connected with other women in the villages.

Social media, online messaging and calling applications were reported to be widely used by VHWs at sites to provide information and basic consultations to community members, including Facebook and Zalo, the most popular social media platform in Vietnam. For those without a smartphone or who are unresponsive to the messages, VHWs described visiting their households in person to provide information, such as dates of vaccination or invitation for oral vitamin delivery for children at the commune health station.

*“I cover several hundred children in the village…within a day*, *how can I distribute all of those invitation letters? Right? I need to distribute them in a few days*. *But now*, *we’re living in the modern time*, *I befriended mothers on Facebook*, *or on Zalo*. *Then whenever I have some notifications*, *I post them online and ask the mothers to visit my home and take the invitation letter*.*”*

*(Female*, *Village health worker 4*, *In-depth interview*, *Binh Thuan)*

*“People see my posts online*; *I announce the schedule for Long Chau village with exact time and date*. *The mothers in here [in her village] are already familiar with that*, *in case they’re still confused*, *they call me*.*”*

*(Female*, *Village health worker 5*, *In-depth interview*, *Phu Yen)*

#### Motivations and performance

The interviews indicated that workers with qualifications and skills were likely to be recruited and motivated to join the taskforce. Five of eight VHWs and one MPW, were trained as nurses or pharmacists at vocational schools. Four VHWs mentioned that the role has helped them gain public health experience and provided them with opportunities when seeking work positions at the CHS or other health facilities. As a shop owner, one female VHW described selling products for pregnant woman and children and perceived that her work as a VHW had helped increase her sales because she could provide reliable information to her clients.

*“The pregnant women here*, *well*, *they know I am working as a village health worker*, *then they usually reach out to me to ask*, *like the schedule of vaccination for children… I am selling diapers also*, *diaper for kids*, *so I need the mothers to support*. *If the kids have fever after getting vaccination*, *the mothers text me and I provide them free consultation*. *That’s why the moms love me*, *they always support me by purchasing stuff I am selling*.*”*

*(Female*, *Village health worker 4*, *In-depth interview*, *Binh Thuan)*

*“I like it here*. *Although I am not a permanent staff working in the government system*, *in the public health system*, *I feel good working here*. *I can apply what I have learnt and my specific skills*. *I have a job and contribute to society*.*”*

*(Female*, *Village health worker 3*, *In-depth interview*, *Binh Thuan)*

Renumeration and workload were seen as key motivating factors for the workers. At the sites, VHWs reported occasions where they received their allowance on a quarterly basis and there were frequent delays in receiving that payment, from a few weeks to months. As workers often worked multiple jobs to secure their living, including their main work in agricultural activities, they felt their role was sometimes a burden during the peak agricultural season. This was especially true for VHWs who oversaw a relatively high number of households or were given excessive tasks; one provided an example of measuring height and weight of nearly 300 children in the village. One VHW planned to resign from her position to focus on her own business and earn more income.

*“I am working as a farmer as well*. *I go to the farm*, *go there*, *then go home*, *then I work some other tasks like this…working on a lot of things but the allowance is very modest*. *Every time*, *like*, *every month*, *I need to take the [WHO growth] chart and check the children [for height and weight]*. *This costs me much time*. *One or two hours per day*.*”*

*(Female*, *Village health worker 1*, *In-depth interview*, *Phu Yen)*

At the same time, VHWs highlighted how opportunities to work and socialize with CHS staff and their communities were their greatest motivation and brought them most pleasure when carrying out their roles. Their participation in monthly meetings, training courses, and the ability to provide health information were perceived by the workers as crucial to receive recognition from the CHS and to build trust from their community.

*“My work*, *like*, *uh*, *I like to go to the CHS for*, *like*, *meetings or reporting*, *I feel good*. *If I don’t come here*, *just stay at home*, *I feel bored*, *very bored*.*”*

*(Female*, *Village health worker 1*, *In-depth interview*, *Binh Thuan)*

Because most workers were selected among, or nominated from, enthusiastic local residents in endemic communities, they did not possess a certificate from a six-month training course (required for VHWs but not for MPWs, CMAT, MEV and MOT) but had gained trust from their community. Thus policymakers and implementers in malaria programmes agreed that they are the key workforce to achieving malaria elimination by promoting positive prevention and care seeking behaviors in their community and minimizing the cultural and language barriers. In addition, as many workers were also forest goers and farmers, they were confident in suggesting practical malaria prevention methods, such as wearing a mosquito net hat to cover their necks and faces, or wearing long-sleeved clothes made of cotton.

*“Our CMATs are local people*, *many of them are among the forest goers and those who sleep in the farm*. *They know very well and keep suggesting to us ways to improve our programme*.*”*

*Female*, *CSO*, *In-depth interview*, *HN07)*

Nevertheless, the current quality of services provided by VHWs was perceived as an area for improvement. Many respondents, including VHWs themselves, suggested that if VHWs were trained more frequently, the workers could provide better quality information about health issues. This is particularly crucial for complicated tasks, such as activities related to blood collection, as pointed out by one FGD respondent who perceived that the VHW lacked sufficient training and skills to perform such tasks.

*“Like some VHWs*, *if they offer to measure blood pressure*, *it’s totally OK*. *But if they ask to collect my blood sample*, *I would say no*. *The VHW only finished fifth grade*; *I don’t trust her*. *Only OK to measure my blood pressure using the monitor*.*”*

*(Female*, *Focus group discussion 2*, *Phu Yen)*

Apart from testing roles, there were concerns regarding the capacity of these cadres and their ability to provide appropriate medical diagnosis and treatment, following the Law on Examination and Treatment (2009). In particular, one central level policymaker suggested that the law should be strictly enforced to prevent non-medical staff, especially the VHWs, from practicing diagnosis and treatment:

*“I do not want village health workers to participate in providing medical diagnosis and treatment because they cannot ensure the quality of care and do not have any qualifications*.*”*

*(Male*, *Ministry of Health*, *HN04)*

However, implementers at the local level and from international and civil society organizations argued that enforcement should be flexible to increase the accessibility and availability of testing and treatment services, especially for malaria, as long as these activities were under strict supervision by CHSs. They mentioned that the volunteers have to travel long distances to respond to some suspected malaria cases. However, due to legal constraints, they cannot perform any tests or treatment. In several cases, they try to convince individuals to seek help from health facilities, but many refuse. Despite these challenges, the volunteers have demonstrated their ability to reach and support hard-to-reach communities through other activities, such as providing health education and personal prevention items like hats, long-sleeve T-shirts, repellents, and treated bed nets.

### The prospects of sustaining their roles and community-based malaria activities

#### Addressing health concerns in communities

Regardless of the concerns about the quality of service provided by the workers, the communities agreed on the need to sustain their roles for testing and providing malaria prevention support. Specifically, community members expected to continue receiving health information as well as personal protection for malaria prevention from the workers in their communities. Their service provision outside of public-office hours was also appreciated by the communities; a FGD respondent, for example, mentioned that she preferred to consult the VHW when the CHS was closed.

Beyond malaria, suggestions from villagers on expanding roles of VHWs included provision of first aid and household visits. The latter were described as essential for the elderly who live alone without family members, for those with severe illness, and for those who only relied on spiritual healers, known as *thay mo*. Suggestion roles related to encouragement to attend or accompanying them to health facilities were mentioned particularly for these population groups.

Many other community members were also positive about receiving simple medical support at their home from workers with a medical background, for example for testing them when they got fever or discussing health-related topics. Although a few respondents described that they only made enough income to pay for their own living costs, other individuals showed willingness to pay a modest compensation for services that were timely or perceived as necessary.

*“They [VHWs] could provide medical examination and treatment for older people who are not able to visit to the commune health station*, *for example*. *We should ask them to visit and examine*, *hmm*, *or*, *like*, *what services*, *to provide IV fluids services at their homes*, *if they (VHWs) have been trained properly*. *The older people can’t visit the health facilities*. *If they could be supported at home by the VHWs*, *it would be helpful*.*”*

*(Female*, *Villager 1*, *In-depth interview*, *Binh Thuan)*

*“VHWs should provide consultation for the community and encourage them to purchase medicine*. *People here*, *they usually have some superstitious rituals (cúng bái*, *a term used to refer to ritual practices to cure illnesses)*, *how could they recover from that*? *They have to visit to the hospital for medical examination and to get medicine*. *But patients here only worry about praying*.*”*

*(Female*, *Villager 3*, *In-depth interview*, *Binh Thuan)*

Support of timely data collection for malaria and other infectious diseases, health promotion and education, and referral and transport to healthcare facilities, were perceived as potential roles for VHWs by policymakers at the national level. Examples that showcased the effectiveness of utilizing community engagement in addressing public health issues were given from the HIV programme and COVID-19 outbreak response, whereby the general public could take a self-test and notify community-based networks for further instructions and assitance with referral if neccesary. However, they prioritized the improvement in quality of care over expanding the roles for diagnosis and treatment services, either for malaria or other illnesses, especially for VHWs who already performed multiple activities. Although policymakers and implementers did not mention additional roles for MPWs, maintaining their roles and adding more intensive malaria-related activities in, for example, active case notification and investigation, foci investigation and vector control activities, were suggested. For the CSO-supported cadres, CSO implementers did not clearly mention future plans to expand the roles of these cadres, except suggestions to maintain the roles of CMAT in health promotion, especially for malaria and non-communicable diseases (NCDs) in the communities and among forest goers with the support of SCDI programmes.

#### Maintaining the capacity

Inadequate training and equipment due to lack of financial resources for, and high turnover of, workers, posed challenges for VHWs and MPWs to perform their tasks well, let alone expand their roles in the future. Support from former workers and colleagues at CHS was reported as crucial for MPWs. Over-the-counter medicines, such as provision of antimalarials in case forest goers had positive malaria tests, was perceived to contribute to VHWs performing case finding activities.

*“I really want more training*, *to build and advance my knowledge*, *then serve the community*.*”*

*(Female*, *Village health worker 3*, *In-depth interview*, *Binh Thuan)*

Although the monthly compensation was perceived as insufficient to support the multiple activities of VHWs, eight VHWs showed willingness to continue and even expand their work and preferred not to receive payment from the villagers even if they were to provide additional services. One VHW suggested that the workers could refer villagers to the CHS or provide oral rehydration solution to villagers free of charge with costs covered by health insurance or from the provincial budget.

*“I just want to be fully responsible for my role as a village health worker*. *I wish my compensation were higher but it should be from governmental support*, *not from the community*.*”*

*(Female*, *Village health worker 4*, *In-depth interview*, *Binh Thuan)*

Despite facing challenges, such as limited support from current policies and the decreased numbers of VHWs in their province, one interviewed VHW who also serves as a village leader expressed his commitment to maintain his VHW responsibilities as a form of community service. This individual recognized and acknowledged the privileges he had received from his roles and placed a high value on paying it back to his community. He understood that his impact on the community through his VHW work was significant and strived to continue supporting as many people as possible, regardless of external constraints.

*“There were 8 VHWs before*, *they (Province) deducted (VHWs in) 3 villages*, *now only 5 villages have VHWs*, *the former VHWs were dissatisfied and upset…I also wanted to resign last time*, *but then I thought I must serve the locals*. *My parents gave me opportunities to learn*, *I had knowledge*, *I need to find a way to serve my community*.*”*

*(Male*, *Village health worker 8*, *In-depth interview*, *Phu Yen)*

The interviewed MPW wanted to maintain his role and expressed confidence in performing additional tests or tasks if training was provided, especially on how to communicate with villagers and convince them to protect themselves and educate them about health. He was also motivated to open a pharmacy to provide consultation and sell medicines in the village, describing that this would be complementary to his role at the malaria post and would help generate additional income for his family.

*“Now*, *well*, *the support I need right now is a training course*. *You know*, *training to advance my knowledge to work better*, *like malaria and how to ask people smoothly about that*. *Or*, *how to help people to take medical examination*, *and professional knowledge about malaria*.*”*

*(Male*, *Malaria post worker*, *In-depth interview*, *Phu Yen)*

### Potential barriers and enabling factors of role expansion

Several factors influencing the potential to expand the roles of workers were reported and they were intertwined. The need to secure funding and allocation of workers and their roles suggest integration of malaria activities into the health system and expansion of roles among malaria-specific cadres into general health services. However, this proposed expansion needs to minimize duplication and consider the complex legal framework. Particularly, the current legal limitations create challenges to serve hard-to-reach populations and other health needs especially in remote communities. Self-testing devices were suggested as a potential solution.

#### Funding and programme management to support role expansion and integration

A lack of funds to maintain community-based health workers was the biggest concern among interviewed participants, especially national policymakers. The current policy issued in 2019 (Decree 34/2019/NĐ-CP (2019)) indicates that only three part-time workers at village level are included in the State annual budget, excluding VHWs and midwifery workers. Hence these workers are expected to receive compensation from the provincial budget or other sources, such as NGOs if workers are associated with such programmes. However, the respondents explained that the policy has not been fully implemented across the country and could jeopardize the current efforts to maintain the roles of VHWs. For the MPWs and CSO-supported networks, the significant deduction of funds from external donors was reported as the most difficult challenge. Policymakers emphasized the need to further advocate for stronger provincial support for VHWs and tap into the provincial funds to sustain them and the other cadres of workers.

*“This will depend on the strategy after eliminating malaria*. *If the funds are cut and we are unable to provide free quick tests and medicine*, *the role of this kind of support team will also be lost*. *Now*, *all of this depends completely on external funds*. *After (achieving) elimination*, *I do not know whether these funds will still be available to maintain the prevention activities*.*”*

*(Male*, *Ministry of Health*, *In-depth interview*, *HN11)*

Apart from allocation of funding, concerns were also raised about whether the expansion of community-based programmes with new additional roles for MPWs and CSO-supported volunteers will overlap with VHWs. Implementers expressed concerns about the ability of local authorities to coordinate and avoid this duplication, and the probable competition among several village-based programmes, for example, between the VHWs and the village midwives.

*“Now there is a trend to assign all the required tasks at the village level to one person only*. *In some villages*, *the village leader might also perform healthcare-related tasks after receiving some training*. *This is not perfect*, *but there is no other way*.*”*

*(Male*, *central level policymaker*, *In-depth interview*, *HN11)*

*“It is very clear that village health workers are entitled to the allowance prescribed by the Decree*, *operating according to the process and regulations of the commune health station and receiving allowance from local funds*. *For other diseases*, *including malaria*, *we will coordinate with other programs such as tuberculosis*, *malnutrition*, *leprosy*, *and population programmes under supervision of CHS in remote areas*. *So there will be no duplication*.*”*

*(Male*, *local level policymaker*, *In-depth interview*, *HN06)*

To ensure that the health services, such as providing malaria services to a large number of households, were provided effectively without overburdening the VHWs, it was suggested that MPWs and CSO-supported workers could assist and provide similar general health services beyond malaria alongside the VHWs in their communities. While this suggests a potential for integrating malaria-specific workers with the local health system, respondents acknowledged that integration would require better coordination between VHWs, MPWs, and CSO-supported programmes to maximize their collective impact and meet the needs of local communities, with investments needed to ensure they receive the necessary supervision from primary healthcare facilities.

*“To maintain the success in provinces where malaria was already eliminated*, *it’s quite challenging*. *There is no reason for the village health workers to continue their malaria tasks separately from other tasks*, *if there is no malaria case in their village*. *So*, *we still have to integrate the communication for malaria in other programmes*, *provide people with knowledge about malaria in other communication programmes*, *for example in nutrition or immunization programmes*. *Or they might have a big communication campaign and integrate all health information to provide it to people at once*.*”*

*(Male*, *central level policymaker*, *In-depth interview*, *HN11)*

Specifically, implementers perceived that this integration of roles among VHWs and MPWs requires not only the implementation guideline from the national programme, but also political and financial advocacy at the provincial level. Despite the process of health system decentralization, some provinces have not fully implemented the regulations and protocols, namely Circular 07/2013/TT-BYT, which is the legal foundation for VHW benefits in addition to specifications of their functions and tasks. To ensure the continuation of MPWs, a local implementer discussed seeking funding from the Provincial People’s Committee to support MPWs in areas with medium to high risks and ongoing transmission. It was also mentioned that, in low endemic areas, malaria services could be performed by VHWs, whereby the VHWs were trained to perform malaria-related activities such as testing with RDT and roles previously performed by MPWs.

*“Currently the government has empowered local authorities to make financial decisions and manage the expenditure*. *But they still need the policy to be there for them to follow*, *or else they would not be able to implement anything*.*”*

*(Male*, *central level policymaker*, *In-depth interview*, *HN01)*

However, maintaining malaria specific skills while the number of local workers with experience and expertise reduced was a challenge to integrate malaria activities into VHW roles and of MPWs and other cadres into the general health system.

*“Areas with malaria hotspots have been well supplied with enough medical staff*, *especially in the remote places*, *but after some time*, *they leave or move to another position*. *We have lost a lot of well-trained staff*.*”*

*(Male*, *CDC*, *In-depth interview*, *Phu Yen)*

Respondents from an international organization and a local CSO also highlighted that this integration will be of benefit by creating stronger links between these workers and the local health structures, increase sustainability and leverage resources for more training.

*“Our approach is that we try our best to link or connect with the local health structure*.

*That’s why we utilize the health mobilizer (referring to village health workers) and the health commune center staff to increase the sustainability because after the end of the project they will still be there*. *They receive the allowance from the government*, *and they will still work on the malaria programme*. *Right now*, *we provided them with enough skills*, *knowledge and practical experience for them to implement the malaria programme*. *When the funds are reduced and the CSO will stop working on malaria due to this*, *they will still be there to maintain services after malaria elimination*.*”*

*(Female*, *international organization programme staff*, *In-depth interview*, *HN08)*

For the CSO-supported health workers, respondents shared their plans to tap into other external sources to fund other health areas in the malaria-free context and in locations where malaria is in significant decline. Respondents highlighted that the programme will secure more donor funding to maintain the CMAT network and address other health needs in the communities. The aim was to equip their cadres with transferable and adaptable skills and knowledge to respond to other needs in the communities beyond malaria.

*“For example*, *we*, *SCDI*, *are committed to improve the life of the poor population in the central highlands*. *That is the reason why we go for malaria there*, *although malaria was not the priority for people there and not the priority for us*, *to be honest*, *if we look at the burden of disease*, *the mortality or the impact on the population*. *But we go there with malaria because it’s an entry point for us to get to the poorest*, *the most marginalized people–the forest goers and the people who sleep at the farm*. *We go there with a much longer-term agenda*. *We know that eliminating malaria in the Greater Mekong Subregion is a public health priority for the world to stop the transmission of drug-resistant malaria*, *to Sub-Saharan Africa for example*. *But for our organization*, *as we prioritize our commitment to the Vietnamese population*, *it’s an opportunity to contribute to the Global Fund but also to find the entry point for this population*.*”*

*(Female*, *CSO*, *In-depth interview*, *HN07)*

#### Challenges in service provision and reaching vulnerable populations

The current legal framework is perhaps the most important barrier emphasized by policymakers in the central government, indicating that the Law on Medical Examination and Treatment issued in 2009 prevents community-based volunteers who are not licensed medical professionals from engaging in any medical diagnostic or treatment activities, even for common diseases. This limits the possibilities of community-based health workers, such as VHWs and CSO-supported workers to provide basic and essential healthcare services, especially in rural disadvantaged areas where medical professionals are most scarce. To address legal constraints on testing service provision by non-medically licensed cadres, self-testing was identified as one of the potential solutions following the successful models of HIV and COVID-19.

*“Now the government is highlighting the social contracting model for community-based organizations to deliver HIV prevention services and community testing*. *We can envision a similar model for other diseases or for primary healthcare in general*. *There is an opportunity there*.*”*

*(Female*, *CSO*, *In-depth interview*, *HN07)*

Although most community members were familiar with the services provided by VHWs and MPWs, it was mentioned that community awareness should be raised for certain activities such as the schedule for child care and vaccination appointments, and the collection of blood samples or testing for malaria. With additional or expanded roles, an introduction and awareness campaign will be needed to facilitate the implementation, particularly for junior workers with less experience, to ensure adherence to follow-up meetings and scheduled consultations. One VHW reported that she was tasked to collect 5–7 blood slides per month for malaria active case detection; however, she was sometimes unable to convince community members to perform fingerstick blood collection and she used her own blood to reach the set quota.

Notably, government and non-governmental implementers had different perspectives regarding the performance of the public health system. Government respondents argued that healthcare coverage and affordability were satisfactory although they acknowledged that further investments were necessary to improve the quality of medical staff and medical equipment at public health facilities. On the other hand, the respondents representing non-governmental organizations maintained that coverage was inadequate, highlighting the need for additional external funding to provide community-based care for hard-to-reach rural populations as well as a tailored health insurance scheme.

## Discussion

Drawing on literature and policy document review and qualitative data, this study underscores the critical role of community-based health workers, namely VHWs, MPWs, and CSO-supported volunteers, in eliminating malaria in Vietnam. Malaria elimination requires sustained efforts over a long period of time, with a strong health care and surveillance system in place to ensure that cases are detected and treated promptly. The involvement of community health workers has been found to be an effective approach to support malaria elimination, especially when they were able to lead and empower the communities and local workers to plan and execute interventions [34]. We found that the workers valued not only the contribution they had from their in the community-based work but also (were motivated by) personal benefits they received such as career advancement or earning money from selling products to their patients. Local volunteers, who often juggle multiple jobs, have the potential to further strengthen the connection between remote communities and the health system, particularly when the workers were professionally trained and wanted to pursue their career progression in public health. This should be encouraged, supported, and recognized with social standing and rewards to maintain community-based care and workforces.

To accompany these endeavors and address local needs, our study found a significant interest in new services, such as data collection, reporting, and support for non-communicable diseases (NCDs), and case referral for hard-to-reach populations. Although implementers perceived that these additional roles could address health priorities in the communities, training the volunteers to deliver additional services in communities while maintaining their malaria specific skills will require continuous investment of time and resources. A lack of financial support to ensure their sustainability and capacity building were seen as the biggest implementation challenges by policymakers and implementers alike, which inevitably results in demotivated workers and undermines their acceptance by the community. Imbalances in workload and renumeration, overlapping roles and competition between community-based volunteers were also emphasized at the national programme level and within the communities. This is consistent with a qualitative study of VHWs in Vietnam, which documented significant challenges in terms of resources, training, and financial incentives for the expansion of VHWs to deliver NCD prevention and control services [35]. These challenges were heavily reported among large-scale community health worker programmes elsewhere [36–38], and especially among lay workers leveraging digital tools in NCD programmes [39]. Many of these were systematic challenges reported in Bangladesh, highlighting that different renumeration structure despite comparable responsibilities demoralized workers, and the importance not only of salaries but also environmental enablers for workers [40].

Effective integration of these health workers into the local health system is crucial for maintaining malaria services, especially to ensure early testing and treatment. However, legal constraints imposed by the Law of Examination and Treatment (2009) [20] limit their ability to provide early diagnostic and treatment services for malaria, resulting in programmes assigning primarily health education and preventive activities to the workers. Although at the time of data collection, treatment provision of any kind was not permissible for VHWs. However, since January 2024, a new mandate according to the Law allows VHWs to provide initial treatment and care for common symptoms and diseases in the community; for example cough, fever, stuffy nose, headache, abdominal pain, upper respiratory tract infection, acute diarrhea, conjunctivitis, flu, dengue fever, typhus, atopic dermatitis, mild allergic hives, measles, mumps, and hand, foot and mouth disease. This new mandate has significant implications for the role expansion strategy as it allows flexibility for VHWs to be tasked with treating roles based on the provincial context.

Self-testing for malaria among hard-to-reach populations was also suggested as a potential intervention by respondents to bypass this legal limitation, and has been shown to be feasible and effective in reaching at-risk groups by an HIV programme piloting and scaling oral fluid-based HIV self-testing in Vietnam [41]. However, a feasibility study of blood-based HIV self-testing, although reporting high acceptance and accuracy of test performance among users, suggested their education level was a critical factor to successfully complete the test [42]. As the malaria RDTs required blood sample collection, this adds complications to enable self-testing among target populations.

Beyond testing and treatment, implementers may also consider the expansion of MPWs and CSO-supported volunteers with similar add-one services to what VHWs could now perform and are tasked with, under the coordination and supervision of local authorities. To accompany this strategy, leveraging domestic funds and resources to maintain these workers was perceived by policymakers and implementers as the optimal option to sustain community-based malaria services. There was also a strong emphasis on setting up a multi-stakeholder collaboration in policy development and implementation, thereby avoiding duplication of existing programmes and effectively managing the networks of community-based workers.

While decentralization offers opportunities for locally tailored interventions, it also brings challenges that have already surfaced regarding lack of capacity building and maintaining support for workers, as evident in the delayed payments for VHWs. Although the National Assembly has requested that each province annually allocates at least 30% of its budget to preventive health measures in 2023, including for malaria [22], 41 provinces have not met this commitment [43]. This deficiency underscores the urgent need for increased awareness at the provincial level regarding the potential resurgence of malaria. Closing this gap would make it more feasible to advocate for increased allocation of funds for malaria prevention in near elimination provinces.

In light of the constrained funding and need to tailor interventions, programmes intending to maintain community-based malaria services and implementing role expansion may consider taking the first step in mapping the priority population groups that are most in need of assistance from VHWs, MPWs and CSO-supported workers, considering the locations with limited access to affordable medical services. The national malaria programme, in particular, may consider developing an awareness campaign, training and supervision sessions, and local advocacy activities, with careful consideration of the legal framework supporting the basic treatment of common symptoms and introduction of new RDTs for community-based testing. Higher-level entities, such as the Ministry of Health, may need to step in to establish specific guidelines and policies to ensure provincial commitment to eliminate malaria by the national target.

## Strengths and limitations

This study contributes to a better understanding of the different types of community-based malaria workers present in the current health system in Vietnam, and perspectives on their current and future roles in the context of malaria control and elimination in Vietnam. We were able to interview multiple key implementers in the malaria programmes at the national and local levels. There were several important limitation to this study, particularly the representativeness of the study participants of the wider situation in these communities and elsewhere in the country. Although policy makers, programme implementers, and managers in the health sector were purposefully selected for interviews as key informants in this study, selection of community members and health workers was based out of commune health stations who are gatekeepers in these communities. This may have resulted in selection bias. Within the study communities, although attempts were made to interview many types of health volunteers, we were able to reach VHWs and one MPW but were not able to recruit CSO-supported volunteers as none were present at the study sites. It is also possible that the views of local healthcare workers and communities in other locations may differ from our findings. However, we were able to collect information regarding the roles of a wider set of workers through interviews with their supervisors and programme managers, and by supplementing this with desk reviews on the programmes managing these workers in the country.

## Conclusion

This study highlights the crucial role of community-based health workers—namely VHWs, MPWs, and CSO-supported volunteers—in eliminating malaria in Vietnam. Achieving this goal requires sustained efforts to maintain accessible case detection and treatment while considering strategies to address local health concerns beyond malaria. Our study found interest in expanding services to include data collection, reporting, support for non-communicable diseases, and case referrals for hard-to-reach populations. Design and implementing these strategies will require prioritizing mapping groups most in need of assistance from these workers and developing contextual enablers to raise community’s awareness and maintain capacity of these workers. Ensuring political advocacy and financial resources to support these workers, as well as coordination between multiple groups, are key to maximizing their impact and ensuring effective integration of malaria activities into the broader health system.

## Supporting information

S1 Checklist Best practices in research reporting checklist. (DOCX)

S1 Appendix In-depth interview and focus group discussion guides. (DOCX)

S2 Appendix The network of malaria programme implementers and cadres of community-based workers in Vietnam. (DOCX)

S3 Appendix Consolidated criteria for reporting qualitative research (COREQ) checklist. (PDF)
