# Supplementary material for: Sustaining village malaria worker programmes with expanded roles: Perspectives of communities, healthcare workers, policymakers, and implementers in Vietnam
Source: PLOS Glob Public Health. 2024 Aug 6;4(8):e0003443. doi: 10.1371/journal.pgph.0003443 (PMC11302919; doi:10.1371/journal.pgph.0003443)
Supplement: S2 Appendix — (DOCX) [file pgph.0003443.s003.docx]

| **Government (Ministry of Health)** | | | **International organisations/ Civil society organisations** | | |
| --- | --- | --- | --- | --- | --- |
| **General health system** | | **Vertical programme** |  |  |  |
| **Central level**   - General Department of Preventive Medicine (GDPM)* - Department of Medical Service Administration (DMSA)* | | - National Institute of Malariology Parasitology and Entomology (NIMPE)* - Institute of Malariology Parasitology and Entomology (IMPE) Quy Nhon and Ho Chi Minh City | - Bill and Melinda Gates Foundation (BMGF) - Clinton Health Access Initiative (CHAI)* - PATH* - President’s Malaria Initiative (PMI)-USAID - World Health Organization (WHO) | | |
|  |  |  | - Provide technical assistance for NIMPE, IMPE or Provincial CDC | | |
| - The GDPM advises and assists MoH in managing and implementing legal regulations in preventive medicine nationwide, including the healthcare workforce. - The DMSA advises the Minister on state healthcare management and implements legal regulations regarding medical services nationwide. | | - NIMPE is responsible for the technical oversight of the national malaria program, which includes developing and reviewing national guidelines, as well as designing, maintaining, and utilizing the surveillance system, with input from IMPEs, provincial CDCs, and technical assistance from external organizations (WHO, PATH, …) | - Health Poverty Action (HPA)* - Vietnam Public Health Association (VPHA) - Population Service International (PSI) - Vietnam Civil Society Consortium for Malaria Control Initiative (VietMCI) - Center for Supporting Community Development Initiatives (SCDI)* | | |
|  |  |  | - Recruit, monitor, provide technical support, and organise capacity building training courses for CSO-based workers - Coordinate and participate in malaria surveillance, investigation, and response activities with local malaria programme - Engage with health facicilities, including both public sector and private sector to manage malaria cases | | |
| **Sub-national level under** the Department of Health (DoH) | | |  | | |
| Provincial Center for Disease Control  Organise active prevention activities and responses to outbreaks, monitor provincial-level implementation | | |  | | |
| District Health Center  Conduct interventions to address epidemics, supervise activities, and monitor malaria situation at district level | | |  | | |
| Commune Health Station  Provide malaria diagnosis and treatment, monitor and supervise treatment adherence | | |  | | |
| Village health worker (VHW)* | Malaria post worker (MPW)* | | Community malaria action teams (CMAT) | Malaria elimination volunteers (MEV) | Mobile outreach team (MOT) |
| - Complete a 6-month training course from the provincial or district levels to address common health needs and provide health advice in communities - Receive monthly allowance of between 15-25 USD for health promotion and prevention outreach activities from the government budget   At sites, VHWs were tasked to perform community health education, health monitoring, activities related to maternal and child healthcare, and participation in health programmes, including malaria related tasks such as prevention promotion, case detection and referral in line with the regulations of the national malaria programme | - Receive training to perform malaria testing and treatment, especially for mobile and migrant populations, and refer severe cases to nearest hospital - Selected from local community members in appropriate area at specific access points such as construction sites and country or forest borders   At sites, MPWs were found to detect and refer suspected cases to CHS, provide rapid malaria tests and blood smears for diagnosis, disseminate malaria prevention information and control vectors by visiting households, and support CHS in detecting, investigating, and reporting cases | | - Perform case detection among mobile and hard-to-reach populations - Refer malaria cases to relevant health facilities including commune health station, mobile outreach teams, or private clinics for testing and treatment Disseminate information using IEC/BCC materials and LLIN/LLIHN to target populations   Conduct surveys and report data via smartphone application | - Perform case detection among mobile and hard-to-reach populations by taking temperature measurement - Refer people at risk of malaria or having malaria symptoms to relevant health facilities including CHS or private clinics for testing and treatment - Disseminate information using IEC/BCC materials and LLIN/LLIHN to target populations   Conduct surveys and report data via smartphone application | - Conduct community-level case detection, diagnosis, treatment and IEC among high-risk groups; follow up malaria cases directly and via VHWs; conduct 5-day outreach activities monthly and map locations of mobile and migrant populations and communities - Implemented in four provinces, including Gia Lai, Dak Lak, Dak Nong and Binh Phuoc; in Binh Phuoc and Dak Nong Provinces, MOT comprised of 2 Commune Health Staff and 1 VHW from villages nearest to outreach area |
| At-risk population and community in malaria endemic areas* | | | | | |
